# Why Tell Children: A Synthesis of the Global Literature on Reasons for Disclosing or Not Disclosing an HIV Diagnosis to Children 12 and under

**DOI:** 10.3389/fpubh.2016.00181

**Published:** 2016-09-08

**Authors:** Beatrice J. Krauss, Susan Letteney, Chioma N. Okoro

**Affiliations:** ^1^School of Public Health, City University of New York, New York, NY, USA; ^2^Department of Social Work, York College, City University of New York, New York, NY, USA; ^3^Public Health Initiative Consultant, Lagos, Nigeria (formerly affiliated with Rogosin Institute, New York, NY, USA)

**Keywords:** HIV, disclosure, stigma, children, global review

## Abstract

While the psychological and health benefits of knowing one’s HIV diagnosis have been documented for adults and adolescents, practice is still in development for younger children. Moderating conditions for whether or not to tell a child he/she has HIV vary by region and local context. They include accessibility of treatment, consideration of HIV as a stigmatizing condition, prevalence of HIV, and an accompanying presumption that any illness is HIV-related, parent or caregiver concerns about child reactions, child’s worsening health, assumptions about childhood and child readiness to know a diagnosis, and lack of policies such as those that would prevent bullying of affected children in schools. In this systematic review of the global literature, we summarize the reasons caregivers give for telling or not telling children 12 and under their HIV diagnosis. We also include articles in which children reflect on their desires for being told. While a broad number of reasons are given for telling a child – e.g., to aid in prevention, adaptation to illness (e.g., primarily to promote treatment adherence), understanding social reactions, and maintaining the child–adult relationship – a narrower range of reasons, often related to immediate child or caregiver well-being or discomfort, are given for not telling. Recommendations are made to improve the context for disclosure by providing supports before, during, and after disclosure and to advance the research agenda by broadening samples and refining approaches.

## Introduction

In countries spread across the globe, mothers, fathers, grandparents, aunts, uncles, sisters, brothers, and foster parents must face telling one or more of the children in their care that the children have a diagnosis of HIV. Their disclosures often occur against a backdrop of multiple family losses (1–3), and in families and communities where not everyone is accepting of those with HIV (4–6). Caregivers may face the disclosure process alone, have the assistance of health-care providers, delegate the task to providers, or be pre-empted by health systems or inadvertent disclosure (e.g., community gossip, visible records, overheard conversations) (7–9). Caregivers face multiple issues during disclosure, anticipated by those with experience with children and with global health issues.

In the opening paragraph of their 1999 recommendations for disclosure of HIV/AIDS diagnoses to children, the American Academy of Pediatrics (AAP) Committee on Pediatric AIDS [(10), p. 164] succinctly states, “Disclosure of HIV infection status to children and adolescents should take into consideration their age, psychosocial maturity, the complexity of family dynamics, and the clinical context.” The World Health Organization [WHO (11), p. 12] in its 2011 guidance on disclosure to children 12 and under provided greater specificity – “children of school age should be told their HIV-positive status; younger children should be told their status incrementally to accommodate their cognitive skills and emotional maturity, in preparation for full disclosure.” The guidance also broadened the AAP statement to include the community context – schools, institutions, and local and national laws and policies. The broader context was deemed important so that disclosure could be addressed in a culturally sensitive manner, and so that laws, policies, and institutional or community cultures that may be damaging (e.g., lack protections against stigmatization of those with HIV) could be changed to foster the well-being of HIV+ children and their caregivers (10). Both the AAP and WHO documents describe disclosure as a process rather than a single event, suggest that HIV+ children’s caregivers may need to be supported in the disclosure process, and underline the well-being of affected children as a fundamental guide to disclosure decision-making.

Several clinicians, researchers, and reviews have suggested that knowing their HIV diagnosis in a timely manner enhances children’s cooperation with antiretroviral therapy (ART) (11–14), helps children understand their illness and promotes self-care (15), relieves a burden of secrecy within families (5), prepares children to protect others as they approach the teen years which often include greater sexual and drug transmission risks (16), and, fundamentally, is a right as children mature (4, 8, 17). Risks of disclosure to children have also been noted (14), but these appear outweighed by benefits (18).

Although progress has been made in reducing HIV prevalence among children, especially in preventing mother to child transmission during pregnancy, childbirth, and through breastfeeding, timely disclosure to HIV+ children remains a concern. At the end of 2014, an estimated 2.6 million children under age 15 were living with HIV worldwide, the majority in low and middle income countries, an estimated 85% in 21 sub-Saharan countries and India. There were approximately 220,000 new infections that year; and only an estimated 32% of children living with HIV or AIDS were accessing ART, much lower than the figures for the adolescent/adult population (10, 19). Worldwide a minority of HIV+ children 12 and under (<5%) have acquired HIV through blood transfusion, needle sticks, sex (sexual abuse, early sexual debut, or sex trafficking), or injected drug use (20); the majority acquired HIV through mother to child transmission (20).

Researchers and clinicians have worked to translate the general guidance for disclosure to children into workable and context-sensitive interventions [e.g., Ref. (3, 9, 21–23)]. However, in the literature to date, it is most often the primary caregiver, with or without assistance from health care workers, who actually discloses to the HIV+ children in their care. In fact, many interventions to aid disclosure, recently developed or under development, begin with interviews of caregivers about the issues facing them around disclosure. The current review synthesizes articles published about caregivers worldwide as they reflect on the reasons they have or have not disclosed to HIV+ children. In Section “Discussion,” the review is expanded by including articles, which summarize the viewpoints of health-care personnel and articles summarizing the reflections of HIV+ children subsequent to their disclosure experiences. Finally, recommendations are made for improving research and practice for disclosure to children 12 and under.

## Methods

### Definition of Disclosure

In 1997, Funck-Brentano et al. (24) proposed patterns of disclosure: (1) full disclosure in which HIV is named as the diagnosis, (2) partial disclosure in which factual discussions are held about symptoms, treatments, and immunodeficiency, and (3) two forms of non-disclosure – avoidance/delay and misnaming of the illness. In our review, we are defining disclosure as full disclosure, thus limiting the number of articles eligible for inclusion.

### Eligibility of Articles

To be included, articles needed to focus on issues regarding disclosure of children’s HIV status to HIV+ children 12 and under. Articles were included if some, but not all, children met the age criteria, i.e., if an article concerned 10–18 year olds. Since ART has had a profound effect on the conception of HIV as a treatable illness, articles prior to 1996, considered the benchmark year for the introduction of ART (21), were excluded. Articles published through December 2015 were included. A subset of eligible articles was entered into a database concerning caregivers’ rationales for disclosing or not disclosing. The remaining articles served as background material to enhance discussion of the database summary and of recommendations concerning disclosure research and practice.

### Eligibility for Inclusion in a Database of Disclosure Rationales

To be included in a database summarizing the reasons caregivers gave for fully disclosing or not fully disclosing, the caregivers had to be discussing an actual, rather than hypothetical, disclosure situation. That is, their descriptions were focused on the child/children they were caring for and why they had or had not told the child his or her HIV status. General discussions about how caregivers ought to behave, what respondents thought guided caregivers in general, or what caregivers said they might do in the future were excluded from the database.

### Search

A search of the 60 international databases of the academic arm of EBSCO^1^ was made using the terms (HIV or AIDS), (disclosure or telling or diagnosis), and (children or adolescents) from January 1996 through December 2015. The search was repeated with the additional terms (reason or rationale or influenc* or decision or factor). Results of the search were checked and expanded by a hand search of references in review articles and by a search of references in key articles. The current search replicated a search conducted by two health librarians, two senior researchers (the first and second authors of the current manuscript) and two research assistants summarizing literature through June 30, 2010 for the WHO guidance on disclosure (18). However, the current search was limited to disclosure of the child’s own HIV status, limited to the post-ART era, and extended to the end of 2015. The current search was conducted by the first and third authors and checked by the second author through hand search of references in key articles and reviews.

### Analysis

Abstracts and Method sections were read to ascertain eligibility. Full text copies of all eligible articles, save one, were obtained. The remaining article was read on-line as full text. Two articles were translated into English from Spanish and from Portuguese sources; the remaining full texts were in English.

All eligible articles were read in full and then summarized, with a subset of articles read by two to three of the authors to confirm the accuracy of the summaries. An Excel database was created for the 18 articles concerning caregivers’ rationales for full disclosure or non-disclosure. It included the source of the sample, data sources (e.g., interview, questionnaire), country in which the research was conducted, descriptors of the total child sample, descriptors of the children told and the caregivers who told, rationales for telling and not telling, additional predictors of disclosure, and notes about special concerns regarding the context or the data. As rationales were accrued, higher order categorizations were made, e.g., reasons related to the child’s illness, to the parent–child relationship. Categories and higher order categories were cataloged in the database. Disagreements between the author/analysts were noted and were resolved by discussion.

## Results

### Search

The full complement of search terms yielded 5,099 articles, the vast majority of which were irrelevant, e.g., hearing “aids.” Removal of articles focused solely on the clinical picture of HIV/AIDS in a pediatric population or treatment of pediatric HIV, disclosure of parental HIV, disclosure in serodiscordant couples, disclosure to family and friends by older youth and adolescents, and disclosure for illnesses other than HIV infection also greatly reduced the number of articles. Of the remaining 147 articles, seven were removed as ineligible (three, although published after January 1, 1996, concerned HIV+ children pre-ART; one was available only as a conference abstract; one concerned HIV+ children above the age range; and one focused solely on the prevalence of mental health issues among HIV+ children with disclosure mentioned as a potential correlate of adjustment). Hand search of the reference sections of review and key articles yielded five additional articles. Thus, the total sample consisted of 145 articles pertinent to disclosure to children with HIV.

### Articles Concerning Caregivers’ Reasons for Fully Disclosing or Not Disclosing

Of these 145 articles, 18 (22, 23, 25–40) were selected as meeting inclusion criteria for this review. Listed in Table 1, articles concerned (1) caregivers’ rationales for fully disclosing and not disclosing children’s HIV status to the children and (2) included HIV+ children 12 and under. Five discussed only reasons for non-disclosure (22, 29, 31, 37, 40); 3 documented only reasons for full disclosure (25, 38, 39); and the remaining 10 covered both full disclosure and non-disclosure (23, 26–28, 30, 32–36). Data originated from 11 countries: 9 of the 22 United Nations priority countries (10) for the elimination of childhood HIV [Democratic Republic of the Congo (DRC), Ethiopia, Ghana, India, Kenya, Nigeria, South Africa, Uganda, and Zambia], and Thailand and the United States of America (USA). Nearly all data were collected from urban, regional, or district hospitals and clinics or networks of clinics through face-to-face interview, or structured, and semi-structured interviews of caregivers, with one study collecting sealed confidential surveys (29). Two investigators (31, 34) purposively collected rural samples. In 7 of the 18 articles, HIV+ children or older youth aware of their status also provided data (26, 28, 30, 35, 38–40).

**Table 1:** **Characteristics of articles 1996–2015 describing caregivers’ reasons for telling or not telling children in their care the children’s HIV diagnoses**.

Reference	Country	Sample source	Source of data	Reasons for disclosing mentioned	Reasons for not disclosing mentioned
Abebe and Teferra (22)	Ethiopia	Pediatric infectious disease clinic associated with a hospital	Open- and close-ended questionnaires for caregivers	–	Yes
Atwiine et al. (23)	Uganda	Tertiary referral pediatric clinic	Structured questionnaire for caregivers and medical records of youth	Yes	Yes
Bhattacharya et al. (25)	India	ART clinic of a North Indian hospital	Semi-structured questionnaire for caregivers and patient records	Yes	–
Boon-Yasidhi et al. (26)	Thailand	Pediatric HIV clinic associated with a hospital	Semi-structured interview of caregivers and youth^a^	Yes	Yes
Brown et al. (27)	Nigeria	University hospital	Semi-structured questionnaire for caregivers	Yes	Yes
Fetzer et al. (28)	Democratic Republic of the Congo	HIV clinic	Semi-structured interview with caregivers and youth^a^	Yes	Yes
Flanagan-Klygis et al. (29)	USA	Hospital-based HIV clinic	Sealed confidential questionnaire for caregivers	–	Yes
Kallem et al. (30)	Ghana	Pediatric HIV clinic	Structured questionnaires for caregivers and youth^a^ and medical records	Yes	Yes
Kiwanuka et al. (31)	Uganda	Rural regional hospital	In-depth interview of caregivers	–	Yes
Lester et al. (32)	USA	Children’s and general hospital	Questionnaires and interview of caregivers	Yes	Yes
Mahloko and Madiba (33)	South Africa	District hospital	Interview of caregivers	Yes	Yes
Mweemba et al. (34)	Zambia	Two clinics and a hospital in a rural area	Case studies and in-depth interview of caregivers	Yes	Yes
Naidoo and McKerrow (35)	South Africa	Pediatric ART clinic associated with a hospital	Questionnaires administered to caregivers and youth^a^	Yes	Yes
Oberdorfer et al. (36)	Thailand	A university and a district hospital	Semi-structured questionnaire for caregivers	Yes	Yes
Tadesse et al. (37)	Ethiopia	Hospital-based HIV/AIDS clinic	Structured questionnaire for caregivers and patient records	–	Yes
Vaz et al. (38)	Democratic Republic of the Congo (DRC)	Three urban care and treatment organizations	Interviews of caregivers and youth^a^	Yes	–
Vaz et al. (39)	Democratic Republic of the Congo (DRC)		In-depth interviews of caregivers and youth^a^	Yes	–
Vreeman et al. (40)	Kenya	Eight HIV treatment health facilities	Baseline questionnaires of caregivers and youth in a disclosure intervention study	–	Yes

*^a^In the indicated articles, youth who already had been told their HIV status were interviewed about the disclosure experience*.

### Disclosure

#### Reasons for Full Disclosure

Reasons that caregivers gave for telling their child(ren) are summarized in Table 2. Thirteen articles examined their reasons for full disclosure (23, 25–28, 30, 32–36, 38, 39). Since these articles varied in whether or not they calculated the frequency with which caregivers used these reasons, the reasons are tabulated according to the number and percentage of articles in which they appeared in tables or text. Heading the list was the “child’s questions or curiosity” (mentioned in 69.2% of the articles). This rationale was followed by “improve adherence” (61.5%), “child age/maturity” (46.2%), “be the one to tell” (46.2%), and “assist child to reduce risks to self and others” (46.2%). Seven additional rationales were, in order of frequency: “child’s right to know” (38.5% of articles), “promote self-care and general health” (38.5%), “keep an honest relationship” (30.8%), “explain disease progression and/or symptoms” (30.8%), “start medication” (15.4%), “explain discrimination” (15.4%), and prepare the child for the disclosure of other’s HIV status, e.g., the status of relatives or friends (7.7%). With the exception of the last two rationales, reasons were distributed across multiple countries. Thus, the rationales appeared to concern maintaining an open parent–child relationship, recognizing the child’s needs and rights, and enlisting the child’s cooperation in promoting health and protecting others.

**Table 2:** **Number of articles citing the specific reasons caregivers offer for disclosing a child’s HIV status to the child**.

Rationale for telling a child his or her HIV status	*Number (%) of articles in which mentioned (N = 13)*	Countries represented	Reference
**Maintain the caregiver–child and other relationships**			
Be the one to tell	6 (46.2)	Uganda	Atwiine et al. (23)
		Thailand	Boon-Yasidhi et al. (26)
		India	Bhattacharya et al. (25)
		S. Africa	Mahloko and Madiba (33)
		Zambia	Mweemba et al. (34)
		DRC	Vaz et al. (38)
Keep an honest relationship	4 (30.8)	Thailand	Boon-Yasidhi et al. (26)
		USA	Lester et al. (32)
		Zambia	Mweemba et al. (34)
		DRC	Vaz et al. (38)
Prepare child for disclosure of others’ HIV	1 (7.7)	USA	Lester et al. (32)
**Child-related reasons**			
Child’s questioning/curiosity	9 (69.2)	Uganda	Atwiine et al. (23)
		Thailand	Boon-Yasidhi et al. (26)
		Nigeria	Brown et al. (27)
		Ghana	Kallem et al. (30)
		USA	Lester et al. (32)
		S. Africa	Mahloko and Madiba (33)
		Zambia	Mweemba et al. (34)
		S. Africa	Naidoo and McKerrow (35)
		DRC	Vaz et al. (38)
Child’s age/maturity	6 (46.2)	Uganda	Atwiine et al. (23)
		Thailand	Boon-Yasidhi et al. (26)
		Nigeria	Brown et al. (27)
		Ghana	Kallem et al. (30)
		S. Africa	Mahloko and Madiba (33)
		DRC	Vaz et al. (38)
Child’s right to know	5 (38.5)	Uganda	Atwiine et al. (23)
		Thailand	Boon-Yasidhi et al. (26)
		Nigeria	Brown et al. (27)
		USA	Lester et al. (32)
		S. Africa	Naidoo and McKerrow (35)
**Social**			
Explain discrimination/stigmatization	2 (15.4)	Thailand	Boon-Yasidhi et al. (26)
		Thailand	Oberdorfer et al. (36)
**Prevention**			
Reduce risks to self and others	6 (46.2)	Uganda	Atwiine et al. (23)
		Thailand	Boon-Yasidhi et al. (26)
		USA	Lester et al. (32)
		S. Africa	Mahloko and Madiba (33)
		DRC	Vaz et al. (38)
		DRC	Vaz et al. (39)
**Illness-related explanations and events**			
Improve adherence	8 (61.5)	Uganda	Atwiine et al. (23)
		Nigeria	Brown et al. (27)
		DRC	Fetzer et al. (28)
		USA	Lester et al. (32)
		S. Africa	Mahloko and Madiba (33)
		Zambia	Mweemba et al. (34)
		DRC	Vaz et al. (38)
		DRC	Vaz et al. (39)
Promote self-care and general health	5 (38.5)	Nigeria	Brown et al. (27)
		India	Bhattacharya et al. (25)
		Ghana	Kallem et al. (30)
		USA	Lester et al. (32)
		DRC	Vaz et al. (38)
Disease progression/symptoms	4 (30.8)	Uganda	Atwiine et al. (23)
		S. Africa	Mahloko and Madiba (33)
		Thailand	Oberdorfer et al. (36)
		DRC	Vaz et al. (38)
Start medication	2 (15.4)	Thailand	Oberdorfer et al. (36)
		DRC	Vaz et al. (39)

#### Who Is Told, When, and by Whom

The 13 studies documenting full disclosure together covered children in the age range of 4–18 years old. The disclosure rate was 31.0% across the 11 (23, 25–28, 31–36) studies for which it could be calculated; less than a third of the 1,168 HIV+ children and youth sampled had been told their diagnosis. The rate varied from a low of 13.5% in a relatively large Nigerian study (27) to a high of 56.7% in a small Zambian study (34).

Calculating across the 12 studies where the mean or median age at disclosure was reported or could be calculated (23, 25–27, 31–36, 38, 39), on average HIV+ children were 10.6 when told their diagnosis. Mean age varied from a low of 8.7 to a high of 15.0 across studies. Many studies did not include measures of range or variability, so it is difficult to infer the youngest and oldest ages at disclosure.

Caregivers were described with varying degrees of specificity (e.g., parent vs. mother or father) study to study. Table 3 suggests that a significant minority of caregivers were grandparents and other relatives and that health-care personnel had taken on an important role in disclosure. Further, at least three studies (32, 36, 38) documented that children may learn about their HIV status inadvertently or by inference (e.g., looking at their medical records, overhearing discussions, or inferring from HIV public service announcements or talk among school friends).

**Table 3:** **How many are told, at what age, and by whom**.

Reference	Total child *N*	Age range of total sample	Mean age (SD) of total sample	*N* (%) of sample told	Age range when told	Mean age (SD) when told	Who told *N* (%)^b^
Atwiine et al. (23)	307	5–17	Median 8	95 (30.9)	–	11.6 (–)	Mother 47 (50.0)
							Other relative 28 (29.8)
							Father 10 (10.6)
							Grandparent 9 (9.6)
Bhattacharya et al. (25)	145	≥5	9.1 (–)	60 (41.4)	≥5	9.1 (1.4)	Parents 51 (85.0)
							Other relative 6 (10.0)
							Health care 3 (5.0)
							Staff
Boon-Yasidhi et al. (26)	96	5–15	8.6 (–)	19 (20.0)	–	9.6 (–)	Mother 7 (36.8)
							Step-parent 5 (26.3)
							Father 3 (15.8)
							Grandparent 3 (15.8)
							Sister 1 (5.3)
Brown et al. (27)	96	6–14	8.8 (2.2)	13 (13.5)	4.5–13	8.7 (2.2)	Mother 6 (46.1)
							Father 3 (23.1)
							Stepmother 1 (7.7)
							Aunt 1 (7.7)
							Grandfather 1 (7.7)
							Health care 1 (7.7)
							Worker
Fetzer et al. (28)	20	9–17	–	4 (20.0)	–	–	Caregiver 3 (75.0)
							Other 1 (25.0)
Kallem et al. (30)	71	8–14	10.4 (1.7)	15 (21.1)	–	11.7 (1.9)	Caregivers 15 (100.0)
Lester et al. (32)	51	6–10	8.3 (–)	22 (43.1)	–	Median 9.7	Caregivers 16 (72.7)
							Child knew 3 (13.6)
							Inadvertent 2 (9.1)
							Health care 1 (4.5)
							Worker
Mahloko and Madiba (33)	149	4–17	8.2 (3.1)	59 (39.6)	4–17	9.3 (2.9)	Mother 23 (39.7)
							Grandparent 17 (29.3)
							Health care 13 (22.4)
							Worker
							Other relative 5 (8.6)
Mweemba et al. (34)	30	10–15	11.9 (–)	17 (56.7)	10–15	12.2 (–)	–
Naidoo and McKerrow (35)	100	8–14	10.9 (–)	27 (27.0)	–	11.6 (0.3)	Parent 23 (84.6)
							Grandparent 3 (11.5)
							Nurse 1 (3.9)
Oberdorfer et al. (36)	103	6–16	9.5 (–)	31 (30.1)	4–15	9.2 (3.0)	Grandparent 10 (32.2)
							Health care 9 (29.0)
							Provider
							Mother 5 (16.1)
							School friends 5 (16.1)
							Other relative 2 (6.4)
Vaz et al. (38)^a^				19	10–18	15.0 (–)	Health care 11 (52.4)
							Worker
							Parent 9 (42.9)
							Inadvertent 1 (14.3)
Vaz et al. (39)^a^				8	8–16.5	12.6 (–)	Mother 5 (71.4)
							Father 1 (14.3)
							Health care 1 (14.3)
							Worker

*^a^Vaz et al. (38, 39) studied only HIV+ youth who had been disclosed to; thus, the proportion who experienced disclosure could not be calculated*.

*^b^In some cases, the number of caregivers is greater than the number of children told, because the youth were told jointly by multiple caregivers; in others, the number of caregivers is less because multiple children were told*.

### Reasons for Not Disclosing

Table 4 summarizes the reasons that caregivers gave for *not* telling their child(ren). Fifteen articles (22, 23, 26–37, 40) discussed these reasons. The most prevalent reasons were “anticipation of the child’s negative psychological reaction” (mentioned in 93.3% of the articles). This rationale was followed by “the child is too young to understand” (86.7%), “the child is unable to keep a secret” (66.7%), “potential social rejection of the child” (60.0%), and “the parent fears anger/blame from the child” (53.3%). Five additional rationales were, in order of frequency: “caregiver feels he/she lacks the skills to communicate HIV status to the child” (40.0% of articles), “parent fears shame/guilt” (20.0%), “parent fears rejection by the child” (20.0%), “parent fears child may reject drugs” (20.0%), and “parent fears questions from the child” (13.3%). The rationales appear to concern potential sociopsychological harm to the child or the family with only one rationale focused on managing the illness.

**Table 4:** **Number of articles citing the specific reasons caregivers offer for *not* disclosing a child’s HIV status to the child**.

Rationale for not telling a child his or her HIV status	Number (%) of articles in which mentioned (*N* = 15)	Countries represented	Reference
**Parent–child relationship reasons**			
Fears anger/blame from child	8 (53.3)	Uganda	Atwiine et al. (23)
		Thailand	Boon-Yasidhi et al. (26)
		Nigeria	Brown et al. (27)
		Uganda	Kiwanuka et al. (31)
		USA	Lester et al. (32)
		S. Africa	Mahloko and Madiba (33)
		Zambia	Mweemba et al. (34)
		S. Africa	Naidoo and McKerrow (35)
Fears rejection by child	3 (20.0)	Thailand	Boon-Yasidhi et al. (26)
		USA	Lester et al. (32)
		Uganda	Kiwanuka et al. (31)
Fears questions from child	2 (13.3)	USA	Flanagan-Klygis et al. (29)
		S. Africa	Mahloko and Madiba (33)
**Child-related reasons**			
Anticipates negative psychological reaction by child	14 (93.3)	Ethiopia	Abebe and Teferra (22)
		Uganda	Atwiine et al. (23)
		Thailand	Boon-Yasidhi et al. (26)
		Nigeria	Brown et al. (27)
		USA	Flanagan-Klygis et al. (29)
		Ghana	Kallem et al. (30)
		Uganda	Kiwanuka et al. (31)
		USA	Lester et al. (32)
		S. Africa	Mahloko and Madiba (33)
		Zambia	Mweemba et al. (34)
		S. Africa	Naidoo and McKerrow (35)
		Thailand	Oberdorfer et al. (36)
		Ethiopia	Tadesse et al. (37)
		Kenya	Vreeman et al. (40)
Too young to understand	13 (86.7)	Ethiopia	Abebe and Teferra (22)
		Uganda	Atwiine et al. (23)
		Thailand	Boon-Yasidhi et al. (26)
		Nigeria	Brown et al. (27)
		USA	Flanagan-Klygis et al. (29)
		Ghana	Kallem et al. (30)
		USA	Lester et al. (32)
		S. Africa	Mahloko and Madiba (33)
		Zambia	Mweemba et al. (34)
		S. Africa	Naidoo and McKerrow (35)
		Thailand	Oberdorfer et al. (36)
		Ethiopia	Tadesse et al. (37)
		Kenya	Vreeman et al. (40)
Child unable to keep a secret	10 (66.7)	Ethiopia	Abebe and Teferra (22)
		Uganda	Atwiine et al. (23)
		Thailand	Boon-Yasidhi et al. (26)
		Nigeria	Brown et al. (27)
		USA	Flanagan-Klygis et al. (29)
		Ghana	Kallem et al. (30)
		USA	Lester et al. (32)
		S. Africa	Mahloko and Madiba (33)
		Thailand	Oberdorfer et al. (36)
		Ethiopia	Tadesse et al. (37)
Potential social rejection of child	9 (60.0)	DRC	Fetzer et al. (28)
		USA	Flanagan-Klygis et al. (29)
		Uganda	Kiwanuka et al. (31)
		USA	Lester et al. (32)
		S. Africa	Mahloko and Madiba (33)
		Zambia	Mweemba et al. (34)
		S. Africa	Naidoo and McKerrow (35)
		Thailand	Oberdorfer et al. (36)
		Kenya	Vreeman et al. (40)
**Caregiver-related reasons**			
Caregiver feels he/she lacks the skills to communicate HIV status to the child	6 (40.0)	Uganda	Atwiine et al. (23)
		Thailand	Boon-Yasidhi et al. (26)
		Uganda	Kiwanuka et al. (31)
		S. Africa	Mahloko and Madiba (33)
		Zambia	Mweemba et al. (34)
		S. Africa	Naidoo and McKerrow (35)
Parent fears shame/guilt	3 (20.0)	Uganda	Atwiine et al. (23)
		Thailand	Boon-Yasidhi et al. (26)
		Zambia	Mweemba et al. (34)
**Illness-related reasons**			
Fears child may reject |drugs	3 (20.0)	Nigeria	Brown et al. (27)
		S. Africa	Naidoo and McKerrow (35)
		Ethiopia	Tadesse et al. (37)

### Correlates of Full Disclosure

It should be noted that for studies that assessed correlates/predictors of full disclosure rarely did the fact that the child was male or female or parent characteristics have an influence. More frequently, significant correlates were related to the child’s illness and health. These included non-perinatal mode of transmission (25); the relative health of the child (feeling well, having hospitalizations, having new symptoms or suddenly worsening health) (35, 36, 40); other health transitions (e.g., starting ART) (25, 33, 40); and experience managing the illness (e.g., longer duration of illness, longer time on ART, self-administration of medicines) (25, 30). Child social and intellectual factors included life transitions (e.g., enrolling in school) (25, 33), and signs of cognitive maturity (being in school, being more educated, higher IQ) (30, 32, 33). For caregivers, both higher (25) and lower education (35) have been associated with disclosure. Disclosure of either the caregiver’s HIV status to the child or the child’s HIV status to other family members (23, 37) have been cited as promoting disclosure. Family communication factors have also been significantly associated with disclosure: children’s persistence in asking questions about their illness (33) and a family environment that encourages the direct expression of feelings (32). Only the presence of the biological father seems to be associated with lower disclosure (30, 33, 36).

## Discussion

Eighteen articles, January 1996 through December 2015, cataloged caregiver’s rationales for fully disclosing or not disclosing children’s HIV status to children 12 and under post-ART. The studies covered an age range for children from 4 to 18, and across 11 countries, including 9 targeted by the United Nations for the elimination of childhood HIV, documented an average rate of disclosure of 31%. On average, HIV+ children who were told were over 10, well beyond the recommendation to tell school-age children (primary school starting ages are typically 5–7 worldwide according to World Bank Data^2^) or the age of assent, 7 years old, for children in clinical trials (18, 41, 42). Indeed some studies did not include children in lower age ranges [e.g., Ref. (32, 39)] restricting the range for mean age of disclosure. A few children learned their HIV status inadvertently; a significant minority was told by grandparents, other relatives or by health-care providers; an apparent majority were told by biological parents.

Although data were gathered principally from urban treatment centers, 69% of the more than 1,100 children and youth in these studies had not learned their HIV status.

Across articles, caregivers cited reasons *for* disclosing that emphasized the child’s needs (the child’s questions or curiosity, child’s age/maturity, child’s right to know, explain discrimination), maintenance of an open caregiver–child relationship (be the one to tell, keep an honest relationship, prepare the child for the disclosure of others’ HIV), and enlisted the child’s cooperation in promoting health and protecting others (improve adherence, assist child to reduce risks to self and others, promote self-care and general health, explain disease progression and/or symptoms, start medication) (23, 25–28, 30, 32–36, 38, 39). In addition, external events, such as enrollment in school (25, 33) or admission to the hospital (35, 36, 40), were cited as motivating disclosure.

Reasons for *not* disclosing concerned fears about potential sociopsychological harm to the child (anticipation of the child’s negative psychological reaction, child is too young to understand, potential social rejection of the child) or to the family (child is unable to keep a secret, parent fears anger/blame from the child, caregiver feels he/she lacks the skills to communicate HIV status to the child, parent fears shame/guilt, parent fears rejection by the child, parent/caregiver fears questions from the child) (22, 23, 26–37, 40) with one rationale focused on managing the illness (caregiver fears child may reject drugs) (27).

Some of these fears have merit as documented by a number of studies. Children’s initial reactions of sadness, worry, confusion, and shock have been described (36, 39, 43), as have instances of discrimination and bullying at school and in communities (1, 8, 44). Caregivers and even health-care providers may lack the knowledge and skills to effectively communicate an HIV diagnosis to the child (45), and some of this may be due to HIV+ caregivers’ own negative disclosure experiences (3).

However, an even broader literature indicates that children’s initial reactions dissipate relatively quickly over time (43, 46) and may be overestimated by caregivers, especially caregivers who themselves have issues of anxiety or depression (35, 47). At least one study, interviewing children, reported that disclosure was not associated with more negative psychological outcomes (40). Knowing one’s HIV status does appear to be associated with stable or improved child well-being (18, 48) and disease management (11–13). Children, themselves, reflecting on disclosure often mention a sense of relief, wishing that disclosure had happened earlier, that their questions had been answered more directly and that more support had been available initially and subsequently in the family, community, and health-care setting (33, 39, 45, 49, 50). In some studies, children also described their own improved adherence (28, 40).

Regarding discrimination, the literature seems to suggest intervention at the local, institutional, and national policy level. It is not only children with HIV who experience teasing and bullying – often especially upsetting to children because of pervasive messages about sexual transmission – but those *presumed* to have HIV because of a rash, an illness, a family illness, slight stature, or geographic location of high HIV prevalence, whether indeed they have HIV or not (1, 6, 44).

### Research Recommendations

#### Include a Broader Set of Populations

In the search for articles worldwide that focused on caregiver reasons for disclosure to children, only 9 of the 22 high priority countries for the elimination of childhood HIV were represented (10). Missing from the 18 articles found were the remaining 13 countries: Angola, Botswana, Burundi, Cameroon, Chad, Cote d’Ivoire, Lesotho, Malawi, Mozambique, Namibia, Swaziland, United Republic of Tanzania, and Zimbabwe. Thus, we do not know if the limited set of rationales, documented here and relatively stable across the countries studied, would also hold for the remaining highly affected countries.

Samples were almost always drawn from urban treatment centers. Many excluded orphans or children at boarding schools. Almost all focused exclusively on perinatally infected children and youth. Thus, our picture of disclosure to children would be enhanced by looking at rural, out-of-treatment, orphaned, boarding school, and non-perinatally infected children as well (22, 31, 51). Some research programs in progress have made steps in that direction (25, 27, 34, 45, 52); some have conducted research at community sites overcoming biases toward studying only treated populations and overcoming barriers to participation in research such as travel to health center research sites [e.g., Ref. (22)].

#### Gather Data in a Way That Enhances Systematic Review or Cross-Study Comparisons

Data standards, such as CONSORT,^3^ recommend collection of summary statistics *and* their precision. That is, statistics ought be reported with accompanying measures of variability; many times, mean age at disclosure was reported without a measure of variability such as the SD; sometimes only frequencies of varying age ranges were reported (e.g., ages 5–9, 10–12, etc.) from which the current authors may or may not have been able to calculate a summary measure such as the median. Standards were often not met regardless of qualitative, quantitative, or mixed-method approaches.

#### Improve Description with More Precise Measures

Articles differed in the precision with which variables were described, e.g., some articles excluded from the current summary did not specify whether the age of the child at the time of disclosure or age at time of interview about disclosure were being discussed. In describing who disclosed, some articles indicated “caregivers” or “health-care personnel,” while others specified the precise relationship of the discloser to the child (e.g., grandfather) or the exact role of the health-care provider (e.g., nurse, infectious disease doctor). Some indicated who was present when a number of individuals participated in disclosing (e.g., multiple people, suggesting some caregivers rely on the simultaneous support of health-care providers and other family members). The greater the precision, the easier it is to make cross-study comparisons and summaries. Further, the literature indicates that health personnel in differing roles may have differing attitudes (34, 53) and that caregivers in differing relationships may have differing issues about HIV disclosure (3, 15). Doctors appear to be greater advocates of early disclosure; HIV+ parents and grandparents may be involved in disclosing their own and relatives’ HIV along with the HIV status of the child (4).

No article asked the caregivers to reflect in a systematic way (e.g., Likert scales, paired comparison) on what experiences or what rationales were most influential in making the disclosure decision. Only one article (29) tried to eliminate the social desirability reporting bias that comes with face-to-face interviews or questionnaires handed directly to study personnel. New techniques, used with low literacy populations, may help reduce such biases by using voice recordings of questionnaires/interviews with study personnel available for assistance.

#### Improve Prediction with Longitudinal Data and Child Well-being Outcome Measures

By definition, the articles summarized here dealt with reflection on past behavior and thus were subject to biases of memory. Most studies relied on caregivers’ recall about date of disclosure, child reactions, and who may have been present. In fact, sometimes HIV+ children’s and caregivers’ accounts did not agree (14, 26, 35). Further, although a rationale may be thought to be influential, until research occurs pre- to post-disclosure, its influence cannot be verified. For example, Jemmott et al. (54), in research on *intention* to disclose found multiple reasons endorsed, but only normative beliefs – the perception that friends, relatives, and others important to the caregiver would want them to tell the child – and self-efficacy – belief they can tell the child – were significant predictors in a multiple regression of the intention to disclose. Further, it is as yet unknown, whether “telling” under certain circumstances or for certain reasons is more or less beneficial for the HIV+ child in physical and mental health domains and in social adjustment.

Longitudinal analysis about illnesses can be exacting (48): over time individuals get better with treatment or decline, older age brings new challenges such as older youth being more involved in maintaining their own treatment, caregiver–child relationships change with time, health-care settings, and personnel change with time. Disclosure unfolds alongside these processes. Documentation over time would inform interventions that could meet time- and maturation-dependent challenges.

#### Transition to Evaluation of Interventions

As suggested by multiple authors (14, 18, 27, 30), the research question no longer seems to be whether or not to fully disclose to children, but when and how. Several promising interventions have been designed or piloted (9, 15, 55, 56), with their common elements described below under clinical recommendations. Nearly all carry with them advice to be age- and context-sensitive.

#### Measure and Report Context at the Individual, Family, Community, Institutional, and National Levels

In largely quantitative articles, however, context is rarely described beyond a few characteristics of the affected children, their caregivers, several attributes of the family situation (e.g., child being raised by relatives, or an HIV+ parent) or where disclosure took place (e.g., home or clinic setting). The articles, providing background for this review, that do describe context – sometimes in case studies, policy papers or research on allied topics such as adherence – are compelling. A few examples will suffice: dilemmas faced by a grandmother who had promised her deceased daughter to never reveal the daughter had died of AIDS, but now discovers the grandchild she is raising is HIV+ (57); disclosure occasioned in a rural district by the child having to travel alone to get care (34); the differing issues for child-headed households in post-genocide Rwanda (8); marginalized and at-risk child and youth populations in India such as street children and children pressed into sex trade (51); secrecy and collusion about illness and medicine-taking within families in a community with high stigmatization of HIV (5); cultural conflict when fathers are family decision-makers, but mothers manage health care and are being told to disclose (15); and the surprise an HIV+ child felt when told her HIV+ status by an apparently healthy HIV+ adult nurse because of the child’s assumptions about how HIV progresses (58). Again some research programs are beginning to assess not only caregiver and health-provider attitudes but attitudes and policies of surrounding communities and institutions such as health-care clinics, hospitals, churches, and schools (1, 7, 8, 52, 54, 59), leading to additional targets for intervention. Systematic reporting about issues at multiple levels will aid context-sensitive full disclosure.

#### Improve Understanding with Mixed Qualitative–Quantitative Research

Several studies summarized here reported comments by older children, caregivers, and health-care providers reflecting on disclosure experiences. Such qualitative data were illuminating. For example, several disclosures were initiated because children did not understand why they had to keep taking medicines if their symptoms had disappeared (28, 34). Importantly, some researches documented that children who had not been told, already knew or suspected their diagnosis (35), or children who supposedly knew had not understood the disclosure fully (26, 40). Children in one study reported having questions post-disclosure while caregivers were unaware of their questions (39). Qualitative data, especially from the viewpoint of the children whose well-being is being fostered, can anticipate and correct likely misunderstandings.

In additional research (50, 60), older youth also indicated where they received valuable support for coping with HIV, described more fully below (see Expand Training before Full Disclosure).

### Clinical Recommendations

Abrupt, delayed, or inadvertent disclosure has been described as harmful (15), while full disclosure has been cited as helpful to HIV+ children (18). As described in Sections “Common Elements in Interventions for Full Disclosure,” “Expand Training before Full Disclosure,” “Expand Support during Full Disclosure,” and “Expand Support after Full Disclosure” interventions are being developed to assist caregivers and children before, during, and after full disclosure so that the potential health benefits of full disclosure can be realized, and potential negative effects anticipated and ameliorated.

#### Recommended Age for Disclosure

While caregivers’ attitudes and beliefs about the best age for disclosure vary widely, systematic guidelines recommend age-appropriate disclosure to school-age children (18). While caregivers hold understandable concerns about potential negative consequences for children of early school age, objective information about children’s short-term reactions such as shock and fear and about long-term benefits such as improved adherence to health regimens may mitigate these worries and provide the impetus for full disclosure. Health-care providers also may often be unaware of best practices in terms of child age; for example, they may lack knowledge of how to use age-appropriate language during full disclosure (9, 15, 55, 56).

#### Common Elements in Interventions for Full Disclosure

Several systematic disclosure interventions have been designed or piloted (9, 15, 55, 56). They have the following common elements: (1) train health-care providers to assist with disclosure, (2) elicit caregiver concerns regarding disclosure, (3) assess child and caregiver readiness for full disclosure, (4) improve readiness by addressing concerns and rehearsing communications to child, (5) disclose with requested assistance, and (6) follow-up with assessment. Generally, the interventions take place in a health-care setting.

#### Expand Training before Full Disclosure

Several articles have suggested the need for training before full disclosure for health personnel, for parents/caregivers, and for children (22, 23, 26–31, 33–35, 37–39). Commonly, pre-disclosure interventions suggest responding to triggers such as questions from the child in honest, age-appropriate ways: for example, explaining about germs and medicines with drawings, and analogies such as the body protecting itself with little soldiers and medicines to help the soldiers (56, 58). Suggestions for health personnel include the use of simple language – terms such as “positive” have a vernacular meaning – and educating caregivers about HIV (57).

However, it also seems to be the case that garnering the community support that will ultimately be needed ought to begin prior to full disclosure [e.g., Ref. (32)]. HIV+ children, ages 10–14, reflecting on their disclosure experiences, commented that safe persons (relatives and friends who knew and understood) and safe places (e.g., a church group) were particularly helpful to them in their adjustment to living with HIV after learning their diagnosis (50). But schools have often been described as not safe. At least one article suggests that few families disclose to school personnel but may need special school services for their HIV+ children (61). It seems reasonable that educative efforts should be extended to these ultimate sources of support, especially since the majority of children in the articles summarized here are in school, some in boarding school separated from family supports (22, 25, 27, 30, 33, 37).

#### Expand Support during Full Disclosure

Blasini et al. (55), in early work, stressed the importance of letting caregivers decide whether they wanted to disclose alone or with the assistance of health-care providers. Preferences in the articles summarized here seem to vary country to country and person to person, with some caregivers wishing to disclose alone, or with other relatives present, or with assistance from health-care providers, or with another complement of individuals (22, 30, 35). Some caregivers wished to defer and let health-care providers disclose directly to the child (7). Sometimes the caregiver decision was co-opted by the health-care system; disclosure occurred without caregiver input [e.g., Ref. (41, 43)]. In some cases, caregivers were actively discouraged from disclosing by health-care personnel (23, 34). Neither article explicated the health-care worker’s reasons for being discouraging, but one documented the caregivers’ strong perception of health personnel’s negative attitudes toward disclosure (34).

Older HIV+ youth reflecting on their earlier disclosure experiences may be an important source of decision-making. In at least one study (62), health-care workers encouraged disclosing alone at home, while youth preferred disclosure with health personnel and the caregiver in a health setting. Youth also often complained that their questions were not heard or answered; communication was directed to adults rather than to them (39). These two studies concern youth who had been told when they were older than early school age. It is likely that preferences might vary by context and child maturity.

Consensus guidelines on breaking bad news in the field of cancer suggest that disclosure should take place across several meetings including enough time to assess the patient’s understanding and emotional status, encourage expression of feelings and respond empathetically, arrange a time to review the situation, offer assistance telling others, and provide information about support services as well as discussing treatment (63, 64). Yet, some caregivers in the studies reviewed here believed disclosure should be a discreet event (31).

#### Expand Support after Full Disclosure

Several articles recommended continuing supports for both caregivers and children post-disclosure (13, 22, 28, 31). Peer groups would assist both caregivers and children to adjust to HIV post-disclosure as would continuing education about HIV and its treatment. Caregivers may need support concerning caregiver–child communication, including appropriate language to use with children, how to explore local cultural factors influencing adjustment to illness, and how to deal with caregiver or child fears about the consequences of disclosure (16). Children may need resources to deal with stigmatizing experiences, to support their physical and mental health, and to aid their access to health care.

### Concluding Statement

The emphasis on whether or not to disclose an HIV diagnosis to children, driving research since the early 1990s, has now shifted to when and how children should be told. Recent evidence suggests that caregivers may want to tell an HIV+ child that the child has HIV, but may fear negative consequences for their families and children. To alleviate their concerns, more support may be necessary prior to, during, and after disclosure. The nature of this support should include the voices of older HIV+ youth aware of their diagnoses, caregivers, health-care providers, and those knowledgeable about local context. To address stigma, support may require changes in institutional policies to address stigmatizing behaviors, encourage physical/mental health, and foster social acceptance in the communities where HIV+ children live. To promote child well-being across the local, institutional, and national contexts that HIV+ children face, future research should aim toward developing “best practice” child-sensitive and context-sensitive standards of HIV disclosure for caregivers and health-care providers.

## Author Contributions

BK and SL made substantial contributions to the conception, design, acquisition of articles, analysis, and interpretation of data for this review. BK and SL had been part of the team that drafted the WHO guideline for disclosure to children 12 and under (18), looking at literature through June 2010 on outcomes for children who had and had not been disclosed to. CO made substantial conceptual and interpretive contributions. For her Capstone Paper in fulfillment of a Master’s Degree in Urban Public Health at City University of New York School of Public Health, Spring 2011, CO focused on the reasons caregivers gave for disclosure within an expanded HIV disclosure literature. The current review updates literature from January 1996 through December 2015 concentrating on caregiver reasons for disclosing and not disclosing to HIV+ children 12 and under. All authors have read, revised, and approved the manuscript, and are accountable for the accuracy of its content.

## Conflict of Interest Statement

The authors declare that the research was conducted in the absence of any commercial or financial relationships that could be construed as a potential conflict of interest.

## Footnotes

^1^https://www.ebscohost.com/academic

^2^http://data.worldbank.org/indicator/SE.PRM.AGES

^3^http://www.consort-statement.org/

## References

[1] Bandason T Langhaug LF Makamba M Laver S Hatzold K Mahere S Burden of HIV among primary school children and feasibility of primary school-linked HIV testing in Harare, Zimbabwe: a mixed methods study. AIDS Care (2013) 25(12):1520–6. 10.1080/09540121.2013.780120 23528004 PMC3898087

[2] Cruz MLS Bastos FI Darmont M Dickstein P Monteiro S The “moral career” of perinatally HIV-infected children: revisiting Goffman’s concept. AIDS Care (2015) 27(1):6–9. 10.1080/09540121.2014.940270 25054808

[3] Galano E De Marco MA Succi RC Silva MH Machado DM. [Interviews with family members: a fundamental tool for planning the disclosure of a diagnosis of HIV/aids for children and adolescents]. Ciên Saúde Colet (2012) 17(10):2739–48. 10.1590/S1413-81232012001000022 23099760

[4] Gachanja G Burkholder GJ Ferraro A HIV-positive parents, HIV-positive children, and HIV-negative children’s perspectives on disclosure of a parent’s and child’s illness in Kenya. PeerJ (2014) 2:e486 10.7717/peerj.486 25071999 PMC4103087

[5] Hejoaka F. Care and secrecy: being a mother of children living with HIV in Burkina Faso. Soc Sci Med (2009) 69(6):869–76. 10.1016/j.socscimed.2009.05.041 19540644

[6] Ishikawa N Pridmore P Carr-Hill R Chaimuangdee K. Breaking down the wall of silence around children affected by AIDS in Thailand to support their psychosocial health. AIDS Care (2010) 22(3):308–13. 10.1080/09540120903193732 20390510

[7] Biadgilign S Deribew A Amberbir A Escudero HR Deribe K. Factors associated with HIV/AIDS diagnostic disclosure to HIV infected children receiving HAART: a multi-center study in Addis Ababa, Ethiopia. PLoS One (2011) 6(3):e17572. 10.1371/journal.pone.0017572 21445289 PMC3061859

[8] Binagwaho A Fuller A Kerry V Dougherty S Agbonyitor M Wagner C Adolescents and the right to health: eliminating age-related barriers to HIV/AIDS services in Rwanda. AIDS Care (2012) 24(7):936–42. 10.1080/09540121.2011.648159 22292484

[9] Boon-Yasidhi V Chokephaibulkit K McConnell MS Vanprapar N Leowsrisook P Prasitsurbsai W Development of a diagnosis disclosure model for perinatally HIV-infected children in Thailand. AIDS Care (2013) 25(6):756–62. 10.1080/09540121.2012.749331 23252607

[10] Joint United Nations Programme on HIV/AIDS (UNAIDs). The Gap Report: Children and Pregnant Women Living with HIV. Geneva: Joint United Nations Programme on HIV/AIDS (UNAIDS) (2014).

[11] Arage G Tessema GA Kassa H. Adherence to antiretroviral therapy and its associated factors among children at South Wollo Zone Hospitals, Northeast Ethiopia: a cross-sectional study. BMC Public Health (2014) 14(1):365. 10.1186/1471-2458-14-365 24735561 PMC4002537

[12] Haberer JE Cook A Walker S Ngambi M Ferrier A Mulenga V Excellent adherence to antiretrovirals in HIV+ Zambian is compromised by disrupted routine, HIV nondisclosure, and paradoxical income effects. PLoS One (2011) 6(4):e18505 10.1371/journal.pone.0018505 21533031 PMC3080873

[13] Polisset J Ametonou F Arrive E Aho A Perez F Correlates of adherence to antiretroviral therapy in HIV-infected children in Lom, Togo, West Africa. AIDS Behav (2009) 13(1):23–32. 10.1007/s10461-008-9437-6 18654845

[14] Vreeman RC Gramelspacher AM Gisore PO Scanlon ML Nyandiko WM. Disclosure of HIV status to children in resource-limited settings: a systematic review. J Int AIDS Soc (2013) 16:18466. 10.7448/IAS.16.1.18466 23714198 PMC3665848

[15] Beima-Sofie K John-Stewart G Shah B Wamalwa D Maleche-Obimbo E Kelley M. Using health provider insights to inform pediatric HIV disclosure: a qualitative study and practice framework from Kenya. AIDS Patient Care STDS (2014) 28(10):555–64. 10.1089/apc.2014.0040 25216105 PMC4183914

[16] Santamaria EK Dolezal C Marhefka SL Hoffman S Ahmed Y Elkington K Psychosocial implications of HIV serostatus disclosure to youth with perinatally acquired HIV. AIDS Patient Care STDS (2011) 25(4):257–64. 10.1089/apc.2010.0161 21323530 PMC3101899

[17] Chew J Beng AL Mun S. Parental concerns about disclosure of a child’s HIV/AIDS status in Singapore. Soc Work Health Care (2012) 51(1):5–21. 10.1080/00981389.2011.622624 22251387

[18] World Health Organization. Guideline on HIV Disclosure Counselling for Children up to 12 Years of Age. Geneva: World Health Organization (2011). 26158185

[19] Joint United Nations Programme on HIV/AIDS (UNAIDS). Core Epidemiology Slides. Geneva: Joint United Nations Programme on HIV/AIDS (UNAIDS) (2015). Available from: http://www.unaids.org/en/resources/documents/2015/20150714_coreepidemiologyslides_ppt 12349391

[20] Sohn AH Hazra R The changing epidemiology of the global paediatric HIV epidemic: keeping track of perinatally HIV-infected adolescents. J Int AIDS Soc (2013) 16:18555 10.7448/IAS.16.1.18555 23782474 PMC3687075

[21] Bartlett JG Ten years of HAART: principles for the future. Paper Presented in Plenary Session at: 13th Conference on Retroviruses and Opportunistic Infections; 2006 Feb 5–8; Denver, Colorado (2006). Available from: http://www.medscape.org/viewarticle/523119

[22] Abebe W Teferra S. Disclosure of diagnosis by parents and caregivers to children infected with HIV: prevalence associated factors and perceived barriers in Addis Ababa, Ethiopia. AIDS Care (2012) 24(9):1097–102. 10.1080/09540121.2012.656565 22316133

[23] Atwiine B Kiwanuka J Musinguzi N Atwine D Haberer JE. Understanding the role of age in HIV disclosure rates and patterns for HIV-infected children in southwestern Uganda. AIDS Care (2015) 27(4):424–30. 10.1080/09540121.2014.978735 25397994

[24] Funck-Brentano I Costagliola D Seibel N Straub E Tardieu M Blanche S Patterns of disclosure and perceptions of the human immunodeficiency virus in infected elementary school-age children. Arch Pediatr Adolesc Med (1997) 151(10):978–85. 9343006 10.1001/archpedi.1997.02170470012002

[25] Bhattacharya M Dubey AK Sharma M. Patterns of diagnosis disclosure and its correlates in HIV-infected North Indian children. J Trop Pediatr (2011) 57(6):405–11. 10.1093/tropej/fmq115 21149240

[26] Boon-Yasidhi V Kottapat U Durier Y Plipat N Phongsamart W Chokephaibulkit K Diagnosis disclosure in HIV-Infected Thai children. J Med Assoc Thai (2005) 88(8):S100–5. 16858851

[27] Brown BJ Oladokun RE Osinusi K Ochigbo S Adewole IF Kanki P. Disclosure of HIV status to infected children in a Nigerian HIV care programme. AIDS Care (2011) 23(9):1053–8. 10.1080/09540121.2011.554523 21476150

[28] Fetzer BC Mupenda B Lusiama J Kitetele F Golin C Behets F Barriers to and facilitators of adherence to pediatric antiretroviral therapy in a Sub-Saharan setting: insights from a qualitative study. AIDS Patient Care STDS (2011) 25(10):611–21. 10.1089/apc.2011.0083 21823909 PMC4530354

[29] Flanagan-Klygis E Ross LF Lantos J Frader J Yogev R Disclosing the diagnosis of HIV in pediatrics. J Clin Ethics (2001) 12(2):150–7. 11642067

[30] Kallem S Renner L Ghebremichael M Paintsil E. Prevalence and pattern of disclosure of HIV status in HIV-infected children in Ghana. AIDS Behav (2011) 15:1121–7. 10.1007/s10461-010-9741-9 20607381 PMC2989337

[31] Kiwanuka J Mulogo E Haberer JE. Caregiver perceptions and motivation for disclosing or concealing the diagnosis of HIV infection to children receiving HIV care in Mbarara, Uganda: a qualitative study. PLoS One (2014) 9(3):e93276. 10.1371/journal.pone.0093276 24667407 PMC3965550

[32] Lester P Chesney M Cooke M Weiss R Whalley P Perez B When the time comes to talk about HIV: factors associated with diagnostic disclosure and emotional distress in HIV-infected children. J Acquir Immune Defic Syndr (2002) 31(3):309–17. 10.1097/00126334-200211010-00006 12439206

[33] Mahloko JM Madiba S Disclosing HIV diagnosis to children in Odi district, South Africa: reasons for disclosure and non-disclosure. Afr J Prim Health Care Fam Med (2012) 2(1):7 10.4102/phcfm.v4i1.34538

[34] Mweemba M Musheke MM Michelo C Halwiindi H Mweemba O Zulu JM “When am I going to stop taking the drug?” Enablers, barriers and processes of disclosure of HIV status by caregivers to adolescents in a rural district in Zambia. BMC Public Health (2015) 15(1):1028 10.1186/s12889-015-2372-3 26445104 PMC4596378

[35] Naidoo GD McKerrow NH Current practices around HIV disclosure to children on highly active antiretroviral therapy. South Afr J Child Health (2015) 9(3):85–8. 10.7196/SAJCH.7957

[36] Oberdorfer P Puthanakit T Louthrenoo O Charnsil C Sirisanthana V Sirisanthana T. Disclosure of HIV/AIDS diagnosis to HIV-infected children in Thailand. J Paediatr Child Health (2006) 42(5):283–8. 10.1111/j.1440-1754.2006.00855.x 16712559

[37] Tadesse BT Foster BA Berhan Y. Cross sectional characterization of factors associated with pediatric HIV status disclosure in Southern Ethiopia. PLoS One (2015) 10(7):e0132691. 10.1371/journal.pone.0132691 26167687 PMC4500496

[38] Vaz L Corneli A Dulyx J Rennie S Omba S Kitetele F The process of HIV status disclosure to HIV-positive youth in Kinshasa, Democratic Republic of the Congo. AIDS Care (2008) 20(7):842–52. 10.1080/09540120701742276 18608054

[39] Vaz LME Eng E Maman S Tshikandu T Behets F. Telling children they have HIV: lessons learned from findings of a qualitative study in sub-Saharan Africa. AIDS Patient Care STDS (2010) 24(4):247–56. 10.1089/apc.2009.0217 20397899 PMC2864057

[40] Vreeman RC Scanlon ML Marete I Mwangi A Inui TS McAteer CI Characteristics of HIV-infected adolescents enrolled in a disclosure intervention trial in western Kenya. AIDS Care (2015) 27:6–17. 10.1080/09540121.2015.1026307 26616121 PMC4685612

[41] Wilfert C Beck DT Fleischman AR Mofenson LM Pantell RH Schonberg SK Disclosure of illness status to children and adolescents with HIV infection. Pediatrics (1999) 103(1):164–6. 9917458 10.1542/peds.103.1.164

[42] Corneli A Vaz L Dulyx J Omba S Rennie S Behets F. The role of disclosure in relation to assent to participate in HIV-related research among HIV-infected youth: a formative study. J Int AIDS Soc (2009) 12:17. 10.1186/1758-2652-12-17 19712468 PMC2753623

[43] Mellins CA Brackis-Cott E Dolezal C Richards A Nicholas SW Abrams EJ Patterns of status disclosure to perinatally HIV-infected children and subsequent mental health outcomes. Clin Child Psychol Psychiatry (2002) 7(1):101–14. 10.1177/1359104502007001008

[44] Zhao G Zhao Q Li X Fang X Zhao J Zhang L. Family-based care and psychological problems of AIDS orphans: does it matter who was the care-giver? Psychol Health Med (2010) 15(3):326–35. 10.1080/13548501003623989 20480436 PMC2879006

[45] Heeren GA Jemmott JB Sidloyi L Ngwane Z Tyler JC. Disclosure of HIV diagnosis to HIV-infected children in South Africa: focus groups for intervention development. Vulnerable Child Youth Stud (2012) 7(1):47–54. 10.1080/17450128.2012.656733 22468145 PMC3314494

[46] Menon A Glazebrook C Campain N Ngoma M. Mental health and disclosure of HIV status in Zambian adolescents with HIV infection: implications for peer-support programs. J Acquir Immune Defic Syndr (2007) 46(3):349–54. 10.1097/QAI.0b013e3181565df0 17721397

[47] New MJ Lee SS Elliott BM Psychological adjustment in children and families living with HIV. J Pediatr Psychol (2007) 32(2):123–31. 10.1093/jpepsy/jsj121 16690751

[48] Butler AM Williams PL Howland DS Hutton N Seage GR Impact of disclosure of HIV infection on health-related quality of life among children and adolescent with HIV infection. Pediatrics (2009) 123(3):935–43. 10.1542/peds.2008-1290 19255023 PMC2697844

[49] Mutumba M Musiime V Tsai AC Byaruhanga J Kiweewa F Bauermeister JA Disclosure of HIV status to perinatally infected adolescents in urban Uganda: a qualitative study on timing, process, and outcomes. J Assoc Nurses AIDS Care (2015) 26(4):472–84. 10.1016/j.jana.2015.02.001 26066697

[50] Phuma-Ngaiyaye EE Dartey AF Experiences of children living with HIV and AIDS following their diagnosis disclosure in Mzuzu, Malawi. Vulnerable Child Youth Stud (2015) 10(4):357–65. 10.1080/17450128.2015.1083639

[51] Naswa S Marfatia YS. Adolescent HIV/AIDS: issues and challenges. Indian J Sex Transm Dis (2010) 31(1):1–10. 10.4103/0253-7184.68993 21808429 PMC3140141

[52] Vreeman RC Scanlon ML Mwangi A Turissini M Ayaya SO Tenge C A cross-sectional study of disclosure of HIV status to children and adolescents in Western Kenya. PLoS One (2014) 9(1):e86616. 10.1371/journal.pone.0086616 24475159 PMC3903588

[53] Watermeyer J ‘Are we allowed to disclose?’: a healthcare team’s experiences of talking with children and adolescents about their HIV status. Health Expect (2015) 18(4):590–600. 10.1111/hex.12141 24112299 PMC5060803

[54] Jemmott J Heeren G Sidloyi L Marange C Tyler J Ngwane Z. Caregivers’ intentions to disclose HIV diagnosis to children living with HIV in South Africa: a theory-based approach. AIDS Behav (2014) 18(6):1027–36. 10.1007/s10461-013-0672-0 24310931 PMC4349627

[55] Blasini I Chantry C Cruz C Ortiz L Salabarria I Scalley N Disclosure model for pediatric patients living with HIV in Puerto Rico: design, implementation, and evaluation. J Dev Behav Pediatr (2004) 25(3):181–9. 10.1097/00004703-200406000-00007 15194903

[56] Trejos AM Reyes L Bahamon MJ Alarcón Y Gaviria G. [Effects in the adherence treatment and psychological adjustment after the disclosure of HIV/AIDS diagnosis with the “DIRE” clinical model in Colombian children under 17]. Rev Chilena De Infectol (2015) 32(4):408–15. 10.4067/S0716-10182015000500007 26436785

[57] Penn C. ‘Too much for one day’: a case study of disclosure in the paediatric HIV/AIDS clinic. Health Expect (2015) 18(4):578–89. 10.1111/hex.12140 24118752 PMC5060797

[58] Nzota MS Matovu JKB Draper HR Kisa R Kiwanuka SN Determinants and processes of HIV status disclosure to HIV-infected children aged 4 to 17 years receiving HIV care services at Baylor College of Medicine Children’s Foundation Tanzania, Centre of Excellence (COE) in Mbeya: a cross-sectional study. BMC Pediatr (2015) 15:81 10.1186/s12887-015-0399-3 26173426 PMC4502565

[59] Mburu G Ram M Oxenham D Haamujompa C Iorpenda K Ferguson L Responding to adolescents living with HIV in Zambia: a social-ecological approach. Child Youth Serv Rev (2014) 45:9–17. 10.1016/j.childyouth.2014.03.033

[60] Petersen I Bhana A Myeza N Alicea S John S Holst H Psychosocial challenges and protective influences for socio-emotional coping of HIV+ adolescents in South Africa: a qualitative investigation. AIDS Care (2010) 22(8):970–8. 10.1080/09540121003623693 20229370 PMC3037257

[61] Mialky E Vagnoni J Rutstein R. School-age children with perinatally acquired HIV infection: medical and psychosocial issues in a Philadelphia cohort. AIDS Patient Care STDS (2001) 15(11):575–9. 10.1089/108729101753287667 11788067

[62] Kidia KK Mupambireyi Z Cluver L Ndhlovu CE Borok M Ferrand RA HIV status disclosure to perinatally-infected adolescents in Zimbabwe: a qualitative study of adolescent and healthcare worker perspectives. PLoS One (2014) 9(1):e87322 10.1371/journal.pone.0087322 24475271 PMC3903632

[63] Ellis PM Tattersall MH. How should doctors communicate the diagnosis of cancer to patients? Ann Med (1999) 31(5):336–41. 10.3109/07853899908995900 10574506

[64] Girgis A Sanson-Fisher RW. Breaking bad news: consensus guidelines for medical practitioners. J Clin Oncol (1995) 13(9):2449–56. 7666105 10.1200/JCO.1995.13.9.2449

